# Germany

**Published:** 1869-04-01

**Authors:** 


### *Germany.*

Appropos of entozoa, Dr. Flamon has reported to the Medical Society of Vienna the outbreak of another epidemic of trichinosis at Shöenbeck (Germany), "that classic land of the trichina." It is asserted that this disease is no longer accidental, but has become actually endemic on the further side of the Rhine.—*Ibid.*

